# No Association of Maternal Gestational Weight Gain with Offspring Blood Pressure and Hypertension at Age 18 Years in Male Sibling-Pairs: A Prospective Register-Based Cohort Study

**DOI:** 10.1371/journal.pone.0121202

**Published:** 2015-03-20

**Authors:** Elina Scheers Andersson, Per Tynelius, Ellen Aagaard Nohr, Thorkild I. A. Sørensen, Finn Rasmussen

**Affiliations:** 1 Department of Public Health Sciences, Child and Adolescent Public Health Epidemiology, Karolinska Institutet, Stockholm, Sweden; 2 Research Unit of Obstetrics and Gynecology, Institute of Clinical Research, University of Southern Denmark, Odense, Denmark; 3 Novo Nordisk Foundation Centre for Basic Metabolic Research, Section on Metabolic Genetics, Faculty of Health and Medical Sciences, University of Copenhagen, Copenhagen, Denmark; 4 Institute of Preventive Medicine, Bispebjerg and Frederiksberg Hospital, Copenhagen, The Capital Region, Denmark; 5 MRC Integrative Epidemiology Unit, Bristol University, Bristol, United Kingdom; University of Alabama at Birmingham, UNITED STATES

## Abstract

**Background:**

Maternal gestational weight gain (GWG) is associated with birth weight, obesity, and possibly blood pressure (BP) and hypertension in the offspring. These associations may however be confounded by genetic and/or shared environmental factors. In contrast to previous studies based on non-siblings and self-reported data, we investigated whether GWG is associated with offspring BP and hypertension, in a register-based cohort of full brothers while controlling for fixed shared effects.

**Methods:**

By using Swedish nation-wide record-linkage data, we identified women with at least two male children (full brothers) born 1982-1989. Their BP was obtained from the mandatory military conscription induction tests. We adopted linear and Poisson regression models with robust variance, using generalized estimating equations to analyze associations between GWG and BP, as well as with hypertension, within and between offspring sibling-pairs.

**Results:**

Complete data on the mothers’ GWG and offspring BP was obtained for 9,816 brothers (4,908 brother-pairs). Adjusted regression models showed no significant associations between GWG and SBP (β = 0.03 mmHg per 1-kg GWG difference, [95% CI -0.08, 0.14], or DBP (β = -0.03 mmHg per 1-kg GWG difference [95% CI -0.11, 0.05]), or between GWG and offspring’s risk of hypertension (relative risk = 1.0 [95% CI 0.99, 1.02], neither within nor between siblings.

**Conclusions:**

In this large sibling-pair study, we did not find any significant association between GWG and offspring BP or the risk of hypertension at 18y, when taking genetic and environmental factors shared within sibling pairs into account. Further large sibling studies are required to confirm a null association between GWG and other cardiovascular risk factors.

## Introduction

It is well known that maternal gestational weight gain (GWG) is associated with a number of pregnancy and birth-related outcomes [1–3]. GWG has in numerous studies been linked to greater adiposity in the offspring in both childhood [4–10] and adulthood [7, 11–13]. From a public health point of view, it is important to determine if the observed association between maternal GWG and offspring adult BMI may also increase cardiovascular risk factors, such as elevated blood pressure (BP). As there may be an underlying association between GWG and BP only in the higher ranges of BP, it is also important to look at the relationship between GWG and hypertension. The majority of studies to date examining the association between higher GWG and BP in the offspring are limited to childhood [14–17]. We are only aware of three studies which have investigated whether GWG also is associated with BP in adulthood [12, 18, 19], and only one of them examined BP classified as hypertension [12]. The results from these studies are inconsistent, with one study finding a weak association between GWG and offspring SBP at 32 years of age [18], while the other two failed to find any statistically significant associations [12, 19].

Due to the scarcity of studies in the area with long follow-up data, the evidence base still remains rather weak. Moreover, and perhaps more importantly, the association between excessive GWG and offspring adult BP in these studies may be confounded by shared familial environmental and/or genetic factors, e.g. socioeconomic status and life-style related characteristics. Family studies provide a way of addressing this type of confounding [20] as parents and their offspring share half of their genes, and so do full siblings on average.

Our objective was to examine the association between GWG and BP, as well as hypertension, in male full sibling pairs at age 18, when genetic and environmental factors fixed from one pregnancy to the next were taken into account.

## Materials and Methods

### Ethics and data availability statement

Ethical approval for the study was granted by the Stockholm Regional Ethical Review Board for the analysis of record-linkage data in the cohort without individual consent (Ref no 2011/691–31/2), in accordance with the Public Access to Information and Secrecy Act and the Personal Data Act. As stated in these regulations, individual consent is not needed when subjects are not actively participating, the information is treated with secrecy, and the results are presented at a group level where no individual is possible to identify. Subject information was anonymized and de-identified prior to analysis. With regards to the data availability, a complete dataset might be generated by Statistics Sweden by record-linkage of data from the public bodies described in the Methods section. Access to the current dataset is restricted due to content of contract (detailed information on data availability can be found in S1 Text).

### Data sources and study population

A database was created for this population-based cohort study by record-linkage of several nation-wide Swedish registers, based on the unique personal identification number assigned to all Swedish citizens. Biological parents were identified from the Multi-Generation Register, where parenthood was established from birth certificates. Although the possibility of false paternity (also referred to as non-paternity) has to be recognized, it has in previous studies been estimated to account for less than 5% in other European countries with similar registers [21, 22]. Information on the mothers’ GWG was retrieved from the Medical Birth Register (which covers 99% of all births in Sweden) while data on BP was collected from the Military Service Conscription Register. We also linked information on level of education from Statistics Sweden’s Register of Education to the database.

### Study sample and exclusions

Data on both exposure and outcome variables was available for all singleton men born in Sweden between 1982 and 1989 (N = 281,522) who underwent military conscription induction tests from 2000 to 2008 (N = 89,829), to which we applied the following exclusions: births to mothers with only one child, mothers with early-pregnancy weight and delivery weight more than or equal to 99 kg (as the MBR had truncated these weights at 99 kg during the years of the study period), gestational age less than 30 weeks or 44 weeks or more and birth weight less than 700 g, as well as systolic BP (SBP) less than 90 mmHg or more than 180 mmHg and diastolic BP (DBP) less than 40 mmHg or more than 100 mmHg (to exclude extreme values due to measurement errors or data entry errors), men in families where only one brother had conscripted, age at conscription less than 17 or more than 20 years, brothers born 3^rd^ or 4^th^ in the cohort (in order to base the analyses on the mothers’ first and second male pregnancies during the study period) and men in families where only one brother had a valid BP measurement. Subsequently, we ended up with a study population of 9,816 full brothers for the main analyses. The data on BP was taken from medical examinations which are part of the military conscription induction tests. Military conscription was compulsory by law for all Swedish men during the period covered in this study, with only 2–3% being exempted due to severe handicaps or chronic disease.

### Measurements of exposure variable and covariates

Maternal GWG, measured as a continuous variable, was the main exposure and was calculated by subtracting the weight at delivery (measured before, and in the same gestational week as delivery) by early-pregnancy weight (measured at the first antenatal clinic assessment, ≈ 10 weeks of gestation). Maternal height and weight (recorded at the first antenatal clinic assessment), parity, birth weight, gestational age, gestational diabetes and preeclampsia were all measured by midwives, obstetricians, or medical doctors as part of normal clinical practice. The quality of the data, in terms of accuracy and completeness, has previously been shown to be “acceptable” (defined as “can be used with some care”) to “good” (defined as “good with a low rate of errors”) [23]. We also collected information from the same register on maternal age at birth, birth year and birth order. According to a report from the National Board of Health of Welfare in Sweden, gestational age at birth has, in the majority of cases, been assessed by ultrasound scans since the 1980’s (with an accuracy of ± 7 days) [24]. Offspring height at conscription was measured using wall-mounted stadiometers, and offspring weight at conscription was measured using analogue or digital scales. BMI (weight in kilograms divided by the square of height in meters) was categorized according to the World Health Organization’s (WHO) classification of BMI cut-offs, into underweight (< 18.50 kg/m^2^), normal (≥18.50 to 25 kg/m^2^), overweight (≥25 to 30 kg/m^2^) or obese (≥30 kg/ m^2^) [25]. Using Statistics Sweden’s Register for Education for the years 1990 to 2010, highest maternal education was categorized into: primary or lower secondary (≤ 10y), secondary (< 12y), full secondary (≥ 12y), higher education < 15y and higher education ≥ 15y.

### Measurement of outcome variables

The main outcomes were BP at conscription (mean age 18.3 years), analyzed as a continuous variable, and the occurrence of hypertension, defined as SBP ≥ 140 mmHg or DBP ≥ 90 mmHg according to the WHO/International Society of Hypertension [26]. The measurements of SBP and DBP were taken after 5 to 10 minutes rest in the supine position with an appropriately sized cuff at heart level, according to a written protocol. The BP was assessed on a single occasion if SBP was 145 mmHg or below and DBP was between 50 and 85 mmHg. However, if SBP and/or DBP were outside these limits, BP was measured a second time on the next day. In these cases, the result of the second BP measurement was entered into the register.

### Sensitivity analyses and handling of missing data

We carried out two separate sensitivity analyses in which we restricted the analyses to 1) offspring born at term (week 37 up to 42 completed weeks) with birth weight of at least 1700 g (according to the criteria for accepted birth weights at gestational week 37, based on a paper by Källén [27], with data from the Swedish MBR on birth weight for gestational age standards) and 2) mothers without diseases during pregnancy (gestational diabetes and preeclampsia). We accounted for missing data in BP and BMI measurements through multiple imputation (MI) in Stata 12.1 using chained equations (20 datasets). However, as the results remained unchanged we only present the results using pairs with complete data (a description and justification of the sensitivity and the MI analyses can be found in S2 Text).

### Statistical analysis

For the main analyses, we used generalized estimating equations (GEE) with robust variance to estimate associations within and between mothers. The regression analyses were performed using the xtgee command in Stata 12.1 (Stata Corp, College Station, Texas, USA). When using BP of the sons as a continuous outcome, the between siblings association was based on the means of GWG in the two pregnancies, and means of the sons’ BP. By contrast, the within siblings association was based on the GWG difference from the mean between two pregnancies of the same mother, and difference in BP between two brothers as outcome. These within and between-analyses are based on the formula: $E\left(Y_{ij\;}\right)=\;\beta_{0}+\;\beta_{w\;}\left(X_{ij}-{\bar{X}}_{i}\right)\;+\;\beta_{B}{\bar{X}}_{i},$ where ${\bar{X}}_{i}$ represents the mean value of *X* for sibling-pair *i* and *β*_*w*_ is the within-pair regression coefficient and *β*_*B*_ is the between-pair regression coefficient. With this analytical approach of differences within full siblings (fixed effects regression), all measured and unmeasured potential confounding factors that do not vary from one pregnancy to the next of the same woman, (e g maternal education, socio-economic status and height) are effectively controlled for [28]. The Wald test was used to test for differences between the within and between regression coefficients. To explore whether differences in GWG were associated with an increased risk of hypertension (dichotomous outcome) in the offspring we also adopted the fixed effects regression design using Poisson regression with robust variance (GEE) to estimate the relative risk (RR).

In order to examine possible underlying associations in sub-groups of the study population, we conducted stratified analyses by the mothers’ early-pregnancy BMI category. As only 6% of the mothers were underweight and less than 4% were obese, these subjects were combined into two categories: “underweight and normal weight (< 25 kg/m^2^)” and “overweight and obese (≥ 25 kg/m^2^)”.

We addressed potential confounding for all types of analyses in three different models. In the basic model (model 1), adjustments were made for maternal age at birth, birth year and gestational age. We then added adjustments for the mothers’ early-pregnancy BMI, parity and maternal education (model 2). In order to account for potential systematic differences between the conscription centers with regards to BP measurements, we further adjusted for conscription center and offspring’s age at conscription (model 3).

## Results

### Descriptive statistics

**Fig 1:**
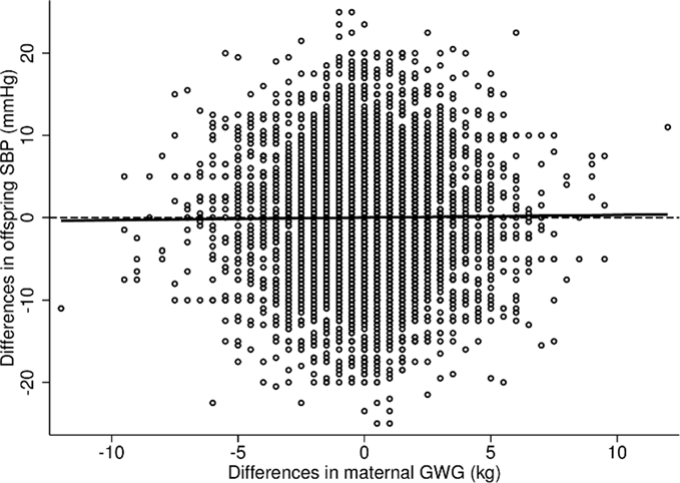
Observed differences in systolic blood pressure and gestational weight gain. The figure shows the unadjusted regression line and observed differences in systolic blood pressure (SBP), mmHg, and gestational weight gain (GWG), kg.

The characteristics of the mothers and their sons, stratified by birth order (first or second born son during the study period), are presented in Table 1. The mean GWG was approximately 14 kg (SD = 4.2 kg), and was slightly lower for the second pregnancy compared to the first (13.8 kg (SD = 4.0 kg) and 14.0 kg (SD = 4.2 kg) respectively). The majority of the sons were normal weight at conscription (79%) and had a mean SBP of 131 mmHg (SD = 11.1 mmHg) and mean DBP of 69 mmHg (SD = 8.4 mmHg). As displayed in Fig. 1, there was a considerable variation in GWG as well as in SBP differences. Additionally, the unadjusted linear regression line, corresponding to the within analyses presented in Table 2, gave no indication of any obvious association between differences in GWG and differences in SBP. The unadjusted prevalence of hypertension was 17% (SBP mean = 146 mmHg (SD = 5.0 mmHg), DBP mean = 75 mmHg (SD = 7.7 mmHg) and was similar across all quintiles of the GWG distribution, although a possible weak trend was observed in the prevalence of hypertension for the second born sons (a table on the prevalence of hypertension across the GWG quintiles can be found in S1 Table).

**Table 1:** Characteristics of the mothers and their sons stratified by birth order.^a^

Characteristics		1st son	2nd son
		(N = 4,908)	(N = 4,908)
Mothers’ characteristics			
	**Early-pregnancy BMI categories [n(%)]**	4,908 (100)	4,908 (100)
	Underweight	346 (7.0)	297 (6.1)
	Normal weight	4,128 (84.1)	4,011 (81.7)
	Overweight	405 (8.3)	546 (11.1)
	Obese	29 (0.6)	54 (1.1)
	**Mean early-pregnancy BMI (kg/m** ^**2**^)	21.6 (2.5)	21.9 (2.7)
	**GWG (kg)**	14.0 (4.2)	13.8 (4.0)
	**Height (cm)**	166.3 (5.8)	166.4 (5.8)
	**Age at birth (y)**	26.7 (3.9)	29.4 (4.0)
	**Highest educational level achieved [n(%)]**	4,908 (100)	4,908 (100)
	Primary or lower secondary ≤ 10 y	348 (7.1)	348 (7.1)
	Secondary < 12 y	1,506 (30.7)	1,506 (30.7)
	Full secondary ≥ 12 y	691 (14.1)	691 (14.1)
	Higher education < 15 y	996 (20.3)	996 (20.3)
	Higher education ≥ 15 y	1,367 (27.9)	1,367 (27.9)
**Sons’ characteristics at birth**			
	**Birth weight (g)**	3595.6 (500.9)	3710.9 (497.3)
	**Gestational age (w)**	39.5 (1.6)	39.5 (1.4)
**Sons’ characteristics at conscription**			
	**Age (y)**	18.3 (0.3)	18.2 (0.3)
	**Weight (kg)**	73.9 (10.9)	74.0 (10.8)
	**Height (cm)**	180.7 (6.4)	180.6 (6.3)
	**BMI (kg/m** ^**2**^)	22.6 (3.0)	22.7 (2.9)
	**BMI category [n(%)]**	4,908 (100)	4,908 (100)
	Underweight	201 (4.1)	160 (3.3)
	Normal weight	3,904 (79.5)	3,882 (79.1)
	Overweight	658 (13.4)	756 (15.4)
	Obese	145 (3.0)	110 (2.2)
	**Systolic BP (mmHg)**	130.6 (11.0)	130.9 (11.1)
	**Diastolic BP (mmHg)**	69.9 (8.4)	68.9 (8.3)

Data are given as mean values (SD) or number of individuals (%). Abbreviations: GWG, gestational weight gain; BP, blood pressure; BMI, Body Mass Index.

^a^ First or second born son during the study period (1982–1989).

**Table 2:** Associations of gestational weight gain with offspring systolic blood pressure, non-stratified and stratified by mother’s early-pregnancy body mass index, using fixed effects regression model.

		Within effect^b^	Between effect^c^	
Statistical model^a^	N	β [95% CI]	β [95% CI]	P-value^d^
**Non-stratified**	9,816			
Model 1		0.03 [-0.06, 0.13]	0.03 [-0.04, 0.09]	0.93
Model 2		0.03 [-0.08, 0.14]	0.02 [-0.05, 0.09]	0.93
Model 3		0.03 [-0.08, 0.14]	0.03 [-0.04, 0.10]	0.95
**Underweight and normal weight**	8,782			
**(BMI < 25 kg/m^2^)**				
Model 1		0.02 [-0.09, 0.13]	0.03 [-0.04, 0.10]	0.86
Model 2		0.02 [-0.10, 0.14]	0.02 [-0.05, 0.10]	0.97
Model 3		0.03 [-0.09, 0.15]	0.03 [-0.04, 0.10]	1.00
**Overweight and obese**	1,034			
**(BMI ≥ 25 kg/m^2^)**				
Model 1		0.13 [-0.13, 0.39]	-0.001 [-0.19, 0.19]	0.40
Model 2		0.06 [-0.25, 0.38]	-0.01 [-0.21, 0.19]	0.67
Model 3		0.03 [-0.27, 0.34]	-0.03 [-0.22, 0.17]	0.75

Abbreviations: CI, confidence interval; BMI, body mass index.

^a^ Model 1 was adjusted for maternal age at birth, birth year and gestational age. Model 2 was adjusted as for model 1 plus early-pregnancy BMI, maternal education and parity. Model 3 was adjusted as for model 2 plus offspring’s age of conscription and conscription center.

^b^ Difference in offspring SBP in mmHg per 1-kg difference in gestational weight gain.

^c^ Difference in offspring SBP in mmHg per 1-kg greater gestational weight gain.

^d^ P-value to test whether the within and between effects differ, obtained by a Wald test.

### Regression analyses

An overall, although weak, association was found between GWG and SBP at 18 years, analyzed with linear regression in the larger cohort (adjusted as per model 3) (N = 89,829) (SBP: β = 0.03 mmHg per 1-kg greater GWG [95% CI 0.01, 0.04], p = 0.001) (DBP: β = -0.01 [95% CI-0.02, 0.0003], p = 0.057). This association might however be confounded by genetic and/or other shared factors, thus motivating further analyses within sibling pairs. No evidence was however found of any association, neither clinically nor statistically significant, between GWG and BP in the offspring at age 18, neither within siblings ((SBP: β = 0.03 mmHg per 1-kg GWG difference, 95% CI [-0.08, 0.14]) and (DBP: β = -0.03 [95% CI-0.11, 0.05])) nor between unrelated families (Tables 2 and 3). Adjustments for potential confounding did not alter the results. We also checked for potential confounding by adding the sons’ concurrent BMI (measured at 18 years) to the third model. As the results were unchanged, and because concurrent BMI could also be a part of the pathway, we chose not to include it in the final model. Moreover, as seen in Tables 2 and 3, the analyses stratified by the mother’s early-pregnancy BMI did not differ from the non-stratified analyses, for neither SBP nor DBP. No association was found between maternal GWG and the risk of hypertension in the offspring (RR = 1.00 [95% CI 0.99, 1.01]) (Table 4).

**Table 3:** Associations of gestational weight gain with offspring diastolic blood pressure, non-stratified and stratified by mother’s early-pregnancy body mass index, using fixed effects regression model.

		Within effect ^b^	Between effect ^c^	
Statistical model ^a^	N	β [95% CI]	β [95% CI]	P-value ^d^
**Non-stratified**	9,816			
Model 1		-0.05 [-0.12, 0.02]	-0.05 [-0.10, 0.004]	0.91
Model 2		-0.03 [-0.12, 0.05]	-0.05 [-0.10, 0.004]	0.79
Model 3		-0.03 [-0.11, 0.05]	-0.04 [-0.09, 0.01]	0.79
**Underweight and Normal weight**	8,782			
**(BMI < 25 kg/m** ^**2**^)				
Model 1		-0.05 [-0.13, 0.03]	-0.05 [-0.10, 0.01]	0.92
Model 2		-0.04 [-0.14, 0.05]	-0.05 [-0.11, 0.01]	0.93
Model 3		-0.03 [-0.12, 0.06]	-0.04 [-0.09, -0.01]	0.84
**Overweight and obese**	1,034			
**(BMI ≥ 25 kg/m** ^**2**^)				
Model 1		-0.03 [-0.21, 0.14]	-0.04 [-0.17, 0.10]	0.97
Model 2		0.01 [-0.22, 0.23]	-0.05 [-0.19, 0.93]	0.69
Model 3		-0.02 [-0.24, 0.20]	-0.06 [-0.19, 0.08]	0.78

Abbreviations: CI, confidence interval; BMI, body mass index.

^a^ Model 1 was adjusted for maternal age at birth, birth year and gestational age. Model 2 was adjusted as for model 1 plus early-pregnancy BMI, maternal education and parity. Model 3 was adjusted as for model 2 plus offspring’s age of conscription and conscription center.

^b^ Difference in offspring SBP in mmHg per 1-kg difference in gestational weight gain.

^c^ Difference in offspring SBP in mmHg per 1-kg greater gestational weight gain.

^d^ P-value to test whether the within and between effects differ, obtained by a Wald test.

**Table 4:** Associations of differences in maternal gestational weight gain with increased risk of hypertension in the offspring at 18 years, using fixed effects regression model (N = 9,816).

	Within effect b	Between effect c	
Statistical model ^a^	RR [95% CI]	RR [95% CI]	P-value ^d^
**Model 1**	1.00 [0.98, 1.02]	1.01 [1.00, 1.02]	0.54
**Model 2**	1.00 [0.98, 1.03]	1.01 [1.00, 1.02]	0.66
**Model 3**	1.00 [0.98, 1.03]	1.01 [1.00, 1.02]	0.61

Abbreviations: RR, relative risk; CI, confidence interval.

^a^ Model 1 was adjusted for maternal age at birth, birth year and gestational age. Model 2 was adjusted as for model 1 plus early-pregnancy BMI, maternal education and parity. Model 3 was adjusted as for model 2 plus offspring’s age of conscription and conscription center.

^b^ Relative risk of hypertension in the offspring per 1-kg difference in gestational weight gain.

^c^ Relative risk of hypertension in the offspring per 1-kg greater gestational weight gain.

^d^ P-value to test whether the within and between effects differ, obtained by a Wald test.

### Additional analyses

In the sensitivity analyses for which we excluded offspring born preterm or post-term (< 37 or > 42 weeks gestation, N = 773), as well as mothers with diseases during pregnancy (gestational diabetes and preeclampsia, N = 82), none of the results differed from those presented earlier and these observations were therefore kept in the analyses. Two additional stratifications were also carried out: in the first one we examined differences in GWG between the two pregnancies. In the second analysis, differences in maternal early-pregnancy weight were considered (a description of the additional stratifications can be found in S3 Text). Similar to the initial stratified analysis, the results from these stratifications did not differ from the main results.

## Discussion

This study investigated the relationship between maternal GWG and offspring BP and the risk of hypertension at 18 years, both within siblings (in order to account for shared environmental or genetic factors) and between unrelated families, however no significant associations were observed.

### Interpretations of main findings

As mentioned briefly in the introduction, only three studies to date have examined the association between maternal GWG and cardiovascular risk factors such as BP in adulthood, with inconsistent results [12, 18, 19], and only one of them analyzed the association of GWG with the risk of hypertension in the offspring [12]. The latter study, based on an Australian birth-cohort of 2,432 individuals, found a modest, although non-significant, association of GWG with SBP at 21 years in the offspring of a sub-group of mothers who had an excessive GWG according to the USA Institute of Medicine guidelines [12]. However, this effect attenuated towards the null in their ‘confounder- and mediator-adjusted model’. The study did not find that adults whose mothers had an excessive GWG had greater odds of hypertension. The other two studies were based on Israeli birth cohorts, one being nation-wide with 10,833 subjects and 17 years of follow-up [19] whereas the other study was restricted to the city of Jerusalem with 1,400 subjects and 32 years of follow-up in the offspring [18]. The smaller of the two studies found a weak, although statistically significant, association between GWG and SBP at 32 years in the offspring. In the larger nation-wide study, no association was found between maternal GWG and offspring SBP and DBP at 17 years and their results were non-significant. As previously discussed, the aforementioned studies were based on unrelated subjects, and the positive findings might therefore be explained by shared environmental and/or genetic factors. Our work extends these previous studies, by taking possible confounding by shared mother-child genetic and environmental factors into account and the results do not suggest any associations between GWG and SBP, DBP or hypertension in young adulthood. As indicated earlier, we found a statistically significant overall association between GWG and SBP in the offspring in the larger cohort of 89,829 men (β = 0.03), but not in the smaller sibling cohort of 9,816 men (β = 0.03 (within and between)). The fact that the effect sizes were similar (albeit small) indicates that the analyses on the sibling cohort may suffer from too low statistical power. As stated briefly in the results section, we also found an indication of a possible trend in increasing prevalence for hypertension with increasing maternal GWG in the second born sons. However, the trend disappeared in the adjusted analysis when we analyzed the association between maternal GWG and the risk of hypertension as described above.

A possible explanation for the lack of association between GWG and young adult BP or hypertension could be that cardiometabolic outcomes such as BP are sensitive to weight gain during specific time windows in gestation. This has been suggested in two large European studies. The first one was conducted on 9-year old children from the United Kingdom where they observed that GWG only during mid-pregnancy (gestational weeks > 14 to 36) was associated with cardiovascular risk factors [16]. The second study, based on 6-year old children from the Netherlands, found an independent association between higher GWG and SBP during both early and mid-pregnancy (gestational weeks > 13.4 to 29.9), although this association was largely mediated by childhood adiposity [29]. Replication of these results are however necessary in studies with longer follow-up periods (into adulthood), but we were not able to do so as our data lacked repeated measurements of GWG.

It is also important to consider the possibility of publication bias, namely that there could be previous studies which have not found any association between GWG and adult BP and/or hypertension, which may not have been published due to the well-known issue that positive findings are more often being published than negative findings. Consequently, the overall understanding of the relationship (or lack of relationship) between GWG and cardiovascular risk factors such as BP, is being compromised.

### Methodological considerations

The major strengths of this study were its prospective design with objectively measured follow-up data in the offspring, availability of data on important confounding factors and its ability to assess the impact of GWG on BP and hypertension while controlling for unmeasured fixed maternal or familial factors within sibling-pairs. These factors include, not only those which do not change from one pregnancy to the next (e g maternal height and possibly diet and lifestyle-patterns), but also those known to affect blood pressure in young adulthood (such as alcohol intake and physical activity [30, 31]) which tend to be more concordant within families (among sibling-pairs) than between families (among non-siblings). Additionally, and in contrast to some earlier studies, both exposure and outcome variables were extracted from national registers and were measured according to a standardized protocol by healthcare professionals, eliminating potential bias from self-reported data.

Our study was also afflicted with some limitations. Firstly, as military conscription tests were only mandatory for men and as the study was conducted in a Swedish population, the results from this study cannot be generalized to women or to ethnic groups other than white European. As previously mentioned, the number of men who completed all the medical examinations involved in the induction tests decreased during the early 2000s, resulting in some missing data for BMI and BP in our cohort. However, as previously acknowledged, the results from the MI analyses suggest that our null-findings were not likely biased due to these missing data.

Although the sibling-design used in our cohort controls for unmeasured shared familial factors, e.g. factors which do not change from one pregnancy to the next, it does not control for unmeasured unshared factors which are pregnancy-specific, e.g. changes in the environment from one pregnancy to the next, such as different dietary and physical activity patterns. In order to limit bias from these unmeasured characteristics, we have however taken confounding factors such as maternal early-pregnancy BMI, age and parity into account in our analyses. It should also be mentioned that there are other unmeasured factors which could influence our outcome differently within the brother-pair (i.e. non-fixed variables, such as history of hypertension or other pre-existing conditions known to affect BP) which we could not take into account in the analyses. However, as these potential confounding variables are likely to create spurious associations (dissimulating true null results), and as we in this study observe null-findings, we believe these factors to be less of an issue. It is further worth pointing out that studies on full-siblings only partially control for genetic confounding, as full brothers on average share half of their parents’ segregating genes. As we only had the possibility to examine one specific cardiovascular risk factor in this study, i.e. BP, and as our study was conducted in 18-year old men where the prevalence of hypertension is lower than in older populations, further large prospective sibling studies with data on other cardiovascular risk factors, as well studies with longer follow-up periods would be worthwhile to conduct.

In summary, the results from our study showed no associations between GWG and BP or the risk of hypertension at 18 years in the offspring when taking fixed genetic and shared environmental factors into account.

## Supporting Information

S1 Table Prevalence of hypertension in the five quintiles of the gestational weight gain distribution. (DOCX)

S1 Text Data availability statement. (DOCX)

S2 Text Description of the sensitivity and the Multiple Imputation (MI) analyses. (DOCX)

S3 Text Description of the additional stratifications. (DOCX)
