# Supplementary material for: No Association of Maternal Gestational Weight Gain with Offspring Blood Pressure and Hypertension at Age 18 Years in Male Sibling-Pairs: A Prospective Register-Based Cohort Study
Source: PLoS One. 2015 Mar 20;10(3):e0121202. doi: 10.1371/journal.pone.0121202 (PMC4368786; doi:10.1371/journal.pone.0121202)
Supplement: S1 Table — (DOCX) [file pone.0121202.s001.docx]

**Table S1.** The unadjusted prevalence of hypertension in the five quintiles (Q) of the gestational weight gain distribution (with the ranges of gestational weight gain of each quintile in brackets), according to birth order (N total = 9,816).

|  | Q1 ((-4) – 10 kg) | Q2 (11-13 kg) | Q3 (14-15 kg) | Q4 (16-17 kg) | Q5 (18-32 kg) |
| --- | --- | --- | --- | --- | --- |
| 1^st^ born (N = 4,908) | 16% | 15% | 16% | 17% | 16% |
| 2^nd^ born (N= 4,908) | 16% | 18% | 18% | 18% | 20% |
