# Supplementary material for: No Association of Maternal Gestational Weight Gain with Offspring Blood Pressure and Hypertension at Age 18 Years in Male Sibling-Pairs: A Prospective Register-Based Cohort Study
Source: PLoS One. 2015 Mar 20;10(3):e0121202. doi: 10.1371/journal.pone.0121202 (PMC4368786; doi:10.1371/journal.pone.0121202)
Supplement: S1 Text — (DOCX) [file pone.0121202.s002.docx]

**Text S1:**

The original data was not collected by the authors, but made available by record-linkage, using the Swedish unique personal ID numbers by the following third parties: Statistics Sweden, The National Archives, The National Service Administration and The National Board of Health and Welfare. The final dataset with the record-linkage was created by a senior statistician (PT) at the Department of Public Health Sciences, Karolinska Institutet (KI). According to a contract signed with Statistics Sweden, the principal investigator (FR) is not allowed to forward the record-linkage dataset with information on individuals, made anonymous by Statistics Sweden, to other researchers outside the Department of Public Health Sciences, KI. Any researcher with approval from the Ethical Review Board in Stockholm is able to create an almost identical dataset by contacting Statistics Sweden (contact person: Andreas Blomquist; andreas.blomquist@scb.se) and the third parties. Statistics Sweden did not agree to save the current dataset with ID numbers for more than three months for correcting any potential errors. The principal investigator will provide metadata (tables) on request to other researchers, for example showing results of alternative analytic strategies.
