# Supplementary material for: No Association of Maternal Gestational Weight Gain with Offspring Blood Pressure and Hypertension at Age 18 Years in Male Sibling-Pairs: A Prospective Register-Based Cohort Study
Source: PLoS One. 2015 Mar 20;10(3):e0121202. doi: 10.1371/journal.pone.0121202 (PMC4368786; doi:10.1371/journal.pone.0121202)
Supplement: S2 Text — (DOCX) [file pone.0121202.s003.docx]

**Text S2:**

**Sensitivity analyses**

The first sensitivity analysis was conducted to examine whether preterm or postterm births (< 37 weeks or > 42 weeks of gestation) could influence the possible association between GWG and BP, and the second analysis was carried out in order to examine whether gestational diabetes (GDM) and preeclampsia (pregnancy-related diseases known to influence GWG [^1^](#_ENREF_1)^,^[^2^](#_ENREF_2)) could confound the results. Due to very few observations on the above mentioned diseases during pregnancy (n = 82), we carried out the second analysis by excluding these observations from the dataset to see whether or not these mothers influenced the results.

**Multiple imputation analyses**

After around year 2000, fewer conscripts needed to carry out all medical examinations due to reorganization of the Swedish Armed Forces. In order to explore potential bias, assuming that BP and BMI measurements were missing at random (MAR), data was also analysed by multiple imputation (MI) in Stata 12.1 using chained equations (20 datasets). These models also included IQ from conscription, parental socioeconomic status and education and father’s BP and BMI from conscription. MI analyses included 1) men who had missing only on the outcome 2) men with missing on both the outcome and predictors and 3) brothers who did not conscript at all.

**Supplemental references**

1. Cedergren M. Effects of gestational weight gain and body mass index on obstetric outcome in Sweden. *International journal of gynaecology and obstetrics: the official organ of the International Federation of Gynaecology and Obstetrics* 2006; **93**(3)**:** 269-274.

2. Hedderson MM, Gunderson EP, Ferrara A. Gestational Weight Gain and Risk of Gestational Diabetes Mellitus (vol 115, pg 597, 2010). *Obstet Gynecol* 2010; **115**(5)**:** 1092-1092.
