# Supplementary material for: No Association of Maternal Gestational Weight Gain with Offspring Blood Pressure and Hypertension at Age 18 Years in Male Sibling-Pairs: A Prospective Register-Based Cohort Study
Source: PLoS One. 2015 Mar 20;10(3):e0121202. doi: 10.1371/journal.pone.0121202 (PMC4368786; doi:10.1371/journal.pone.0121202)
Supplement: S3 Text — (DOCX) [file pone.0121202.s004.docx]

**Text S3:**

We also conducted additional stratifications on two separate subpopulations, to consider potential underlying effects in these subgroups for the associations between GWG and SBP/DBP in the offspring. Firstly, we looked at differences in GWG between the two pregnancies and compared the group of mothers who had a lower GWG versus the mothers who had a higher GWG during the second pregnancy compared to the first. Secondly, we also examined differences in maternal early-pregnancy weight by comparing the group of mothers who had a lower weight versus the mothers who had a higher weight at the beginning of their second pregnancy compared to their first. The results from these stratifications did however not differ from the main findings.
